# The Microstructure, Crystallization Behavior, and Mechanical Performance Evolutions of Li_2_O-Al_2_O_3_-SiO_2_ Glass and Glass–Ceramics with Different Alkaline Earth Oxide Modifications

**DOI:** 10.3390/ma18061383

**Published:** 2025-03-20

**Authors:** Chi Zheng, Danni Li, Mengshuo Guo, Jihong Zhang, Jun Xie, Jianjun Han

**Affiliations:** 1 State Key Laboratory of Silicate Materials for Architectures, Wuhan University of Technology, 122 Luoshi Road, Hongshan, Wuhan 430070, China; zc0523@whut.edu.cn (C.Z.); 281054@whut.deu.cn (D.L.); 330932@whut.deu.cn (M.G.); hanjj@whut.edu.cn (J.H.); 2 Sanya Science and Education Innovation Park of Wuhan University of Technology, Yazhou District, Sanya 572025, China; 3 Hepu Research Center for Silicate Materials Industry Technology, 27 Huanzhu Avenue, Hepu County, Beihai 536100, China

**Keywords:** Li_2_O-Al_2_O_3_-SiO_2_ glass, alkaline–earth oxide, microstructure, glass–ceramics

## Abstract

The introduction of alkaline earth oxides into Li_2_O-Al_2_O_3_-SiO_2_ glass components can improve the mechanical and optical performances of glass and glass–ceramics for various applications. In this research, microstructures, thermal properties, crystallization behavior, and mechanical performance changes in specific Li_2_O-Al_2_O_3_-SiO_2_ glass with the introduction of different alkali earth oxides, MgO, CaO, SrO, and BaO, were investigated. From Raman and NMR spectra microstructure analysis, it was confirmed that the addition of MgO could compete with Al as a network former and charge compensator, while increasing the bridging oxygen number with Si and affecting the chemical shift in ^29^Si. Meanwhile, the glass structures slightly changed due to the introduction of CaO, SrO, and BaO, with larger ionic radii. Meanwhile, the glass transition and first crystallization temperatures increased due to MgO introduction and then decreased with larger-radii alkali earth oxides’ addition, due to different glass network connectivity. After heat treatment, the crystal phases in the glass–ceramics changed with the introduction of alkaline earth oxides. The main crystal phases varied from Li_2_Si_2_O_5_, SiO_2_, and LiAlSi_4_O_10_ in glass without alkali earth oxide introduction; to SiO_2_, Li_x_Al_x_Si_3−x_O_6_, and MgAl_2_Si_4_O_12_ in glass with MgO addition; to SiO_2_ and Li_x_Al_x_Si_3−x_O_6_ with CaO addition; to SiO_2_, LiAlSi_4_O_10_, and Li_2_SiO_3_ for glass with SrO addition; and further to Li_2_SiO_5_, SiO_2_, and LiAlSi_4_O_10_ for glass with BaO addition. Moreover, in the mechanical performance of the glass–ceramics, the Vickers hardness and elastic modulus reached a maximum of 8.61 GPa for glass with MgO and 90.12 GPa for glass with BaO modification, respectively, probably due to different crystal phases. More importantly, the crack resistance values presented a large increase for MgO glass and MgO- or CaO-modified glass–ceramics.

## 1. Introduction

It has long been known that lithium–aluminum–silica glass and glass–ceramics have excellent mechanical properties, significant chemical stability, high thermal shock resistance, low thermal expansion, and high transparency [1,2,3] and are used in mobile device screen protectors, camera lenses for dental applications, and mirrors [4,5,6,7,8,9,10,11,12]. These related mechanical performances can be further enhanced through crystallization processes, crystal phases, and glass compositions [13,14].

Different protocols have been developed to control crystal precipitation and improve mechanical performance. Recently, Fabris et al. [15] surface-crystallized Li_2_O-Al_2_O_3_-SiO_2_-K_2_O-Na_2_O-MgO-CaO glass, increasing the hardness of the crystalline glass from 6.3 GPa to 7.5 GPa, and the optimum fracture strength reached 680 MPa. Yang et al. [16] optimized Li_2_O-Al_2_O_3_-SiO_2_-K_2_O-ANa_2_O-MgO-CaO glass by optimizing the heat treatment process; transparent surface-crystallized aluminosilicate glass with ultra-high C_R_ (75.1 N) and high hardness (7.1 GPa) was prepared. So-Won Kim [17] et al. found that the substitution of Li_2_O with MgO in LAS glass showed the most favorable properties, including a low dielectric constant and low thermal expansion, due to their different alkali earth oxides in the glass investigation. In a study by Weigel et al. [18], the incorporation of Mg, Ca, Sr, and Ba into XAlSiO_4_ glass systems revealed a proportional increase in the atomic packing density and elastic modulus with cation field strength. In general, the introduction of alkaline earth cations into silicate glass can destroy the bridging oxygen bond and glass network connectivity [19], further having an obvious effect on the glass microstructure, high-temperature viscosity, hardness, and modulus and other properties. It is necessary to study the detailed influence of different alkali earth oxide additions on LAS glass, especially on the mechanical properties.

In the current research, different alkali earth oxides, MgO, CaO, SrO, and BaO, were introduced into LAS glass and glass–ceramics with specific compositions. The effects of the alkali earth oxides on the microstructure, thermal properties, and mechanical properties were investigated. In combination with X-ray diffraction (XRD) and scanning electron microscopy (SEM) images, the composition and morphology of the glass crystalline phases were determined to explore the crystallization mechanism. In addition, the mechanical performances were evaluated by the Vickers hardness, elastic modulus, and crack resistance.

## 2. Experimental Section

### 2.1. Glass and Glass–Ceramics’ Preparation

Raw material powder, lithium carbonate (Li_2_CO_3_, ≥98%), alumina (Al_2_O_3_, 99.0%), silicon oxide (SiO_2_, 99.0%), magnesium oxide (MgO, 98.5%), calcium carbonate (CaCO_3_, 99%), strontium carbonate (SrCO_3_, 99%), barium carbonate (BaCO_3_, 99%), zirconium oxide (ZrO_2_, 99%), and antimony trioxide (Sb_2_O_3_, 99.0%) were purchased from the China National Pharmaceutical Group Chemical Reagent Co. Glass specimens with nominal compositions of 70.0SiO_2_-4.3Al_2_O_3_-16.1Li_2_O-2.8ZrO_2_-0.9P_2_O_5_-5.36 RO (mol%) and one additional reference glass without alkaline earth oxide addition were prepared by the conventional melt-quenching method, named LAS, MLAS, CLAS, SLAS, and BLAS, respectively, with RLAS denoting all alkali earth oxide-modified glass. The raw material powders were weighed, mixed thoroughly, melted in a platinum crucible at 1600 °C for 3 h, and annealed in an annealing furnace at 535 °C for 10 h to remove internal stresses. The obtained glass and glass–ceramics were cut into 10 mm × 10 mm × 2 mm dimensions and polished for further characterization.

### 2.2. Characterization

The amorphous state of the prepared samples and the crystalline-phase glass–ceramics were characterized in the range of 10–80° 2θ with a scanning speed of 2°/min and a step size of 0.02° using an X-ray diffractometer (Bruker, D8 Advance, Rheinstetten, Germany). The density of the glass specimen was measured according to Archimedes’ principle. The data were taken from the average of 10 repeated measurements. The molar volume was calculated from the molar weight and density of the glass.

The Raman spectra were collected from an LABHRev-UV laser confocal micro-Raman spectrometer (Horiba, Kyoto, Japan), under a 10.6 mW 532 nm laser beam excitation, with 4 s accumulation duration. The network structure of the glasses was analyzed using a magic-angle spinning NMR spectrometer (MAS-NMR, VANCE III 400, Bruker, Germany). The glass samples were ground into fine powder and then measured at chemical frequencies of 104.26 MHz and 79.49 MHz for ^27^Al and ^29^Si data, respectively. The pulse sequences were single pulses with relaxation delays of 2 and 5 s, respectively.

The glass transition temperatures (T_g_) and crystallization temperatures (T_c_) were measured using a TG-DSC thermal analyzer (STA4F3, NETZSCH, Selb, Germany), with a 25~1000 °C temperature range and a heating rate of 10 °C/min under an ambient atmosphere. Glass rods with dimensions of 5 mm × 25 mm × 5 mm were fabricated to measure the coefficient of thermal expansion (CET) using a thermal expansion meter (DIL 402SE, NETZSCH, Germany).

Samples of glass etched in 10 vol% HF solution for 30 s were made and the crystal morphology in the glass was observed using a field emission scanning electron microscope (FE-SEM, S-4800, Hitachi, Tokyo, Japan) with an accelerating voltage of 5 kV and a beam current of 10 mA.

The Vickers hardness of the glass and glass–ceramics was measured using a microhardness tester (HV-1000A, Huayin Testing Instruments, Guangzhou, China) at a load of 200 g for 10 s. Hardness values were derived from the average of 10 indentations and the corresponding calculated hardness data with an error of 0.2 GPa. The reduced modulus of elasticity was tested using a nanoindentation tester (Bruker hysitron, ti-980).

Thirty indentation angle cracks were generated by optical microscopy at room temperature. The probability of crack initiation was then calculated by dividing the number of radial corner cracks by the total number of indentation angles (four times the number of indentations). The probability of crack initiation was ensured to increase gradually by increasing the indentation load from 0% to 100%. The percentage of crack initiation was plotted against the applied load. A 50% probability load was identified as C_R_, as defined by Wada [20]. The uncertainty of C_R_ was approximately ±1.7 N. All the measurements were performed at room temperature.

## 3. Results and Discussion

### 3.1. The Basic Physical Properties and Microstructures of the Glass

**Figure 1:**
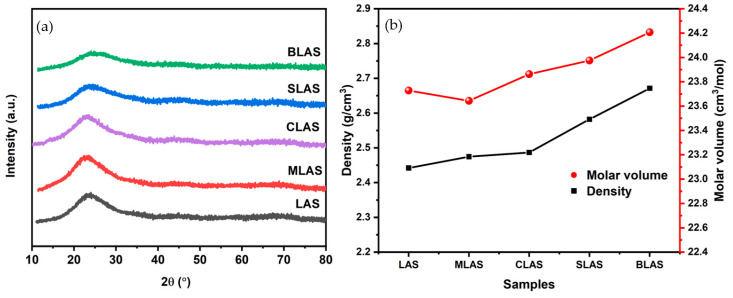
The XRD patterns (**a**) and the densities and molar volumes of LAS and RLAS glass (**b**).

The broad peaks at 20–30° in the XRD patterns of all obtained specimens (Figure 1a) indicated the glassy status. The glass densities gradually increased from 2.44 g/cm^3^ for LAS to 2.67 g/cm^3^ for BLAS (Figure 1b) due to alkali earth oxide addition and larger atomic mass. In addition, the molar volumes of all glasses also increased, which was consistent with B_2_O-Al_2_O_3_-SiO_2_ glass with different alkali earth oxide modifications [21], due to the balance of the growing density and weakened glass structure induced by decreased ion field strength with larger ionic radii of Mg^2+^ to Ba^2+^ ions.

**Figure 2:**
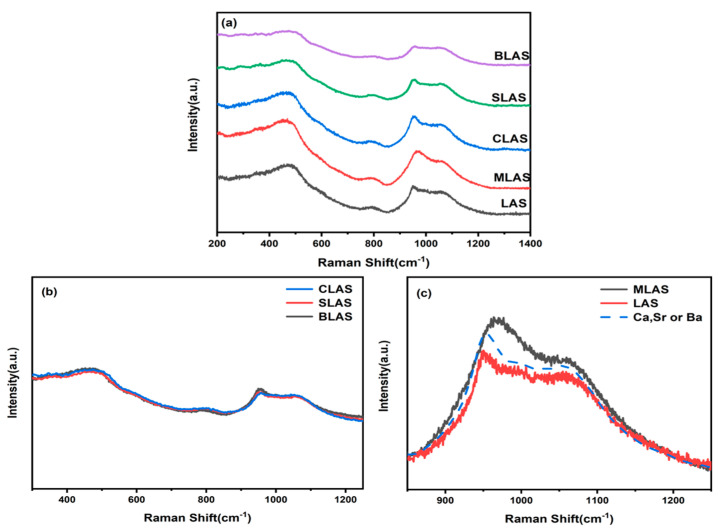
The Raman spectra of the LAS and RLAS glasses. The spectra are shifted vertically for clarity (**a**). Normalized Raman spectra in the range of 300–1250 cm^−1^ (**b**) and in the range of 850~1250 cm^−1^ (**c**).

The Raman spectra of the glasses (Figure 2a) showed three main bands, located at 400–550 cm^−1^, 750–840 cm^−1^, and 900–1200 cm^−1^, which could be attributed to the [Si-O-Si] bending vibration of three or four silica rings in the glass structure, the symmetric stretching vibration of [Si-O-Si] or [Si-O-Al] in the aluminosilicate glasses, and the Si-O stretching vibration, respectively [22,23]. There was no significant microstructure change in the R = Ca, Sr, and Ba glasses, compared with the LAS glass (Figure 2a, inset). The high-frequency band could be attributed to the tensile vibration of T-O (T = Si, Al) in different Q_n_-TO_4_ (n is the bridge oxygen number of the structural unit; Q is Al or Si) tetrahedral units [24,25]. The results indicated that the larger divalent cations (Ca, Sr, or Ba) did not disturb the bridging oxygen in the [SiO_4_] and [AlO_4_] network formation and acted as charge balances. On the other hand, the glass microstructure presented obvious changes due to MgO addition. In fact, the high-frequency band of 850–1250 cm^−1^ was broader and higher. The peak at 850–1250 cm^−1^ was related to the vibration of the glass network structure. This result further confirmed that Mg^2+^ competed with Al^3+^ in the glass structure and ended up acting as a network former [26] and charge compensator [27] to further affect the crystallization and mechanical properties of the glass.

**Figure 3:**
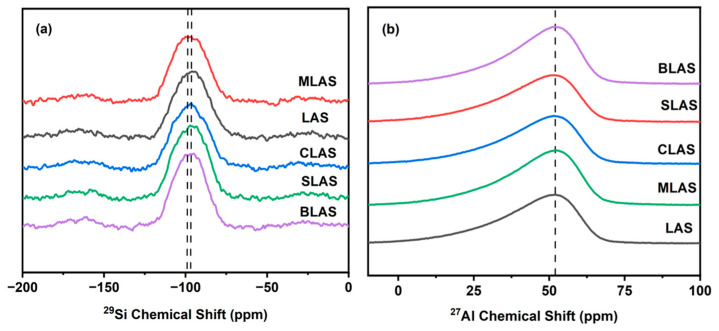
^29^Si MAS NMR spectra (**a**) and ^27^Al MAS NMR spectra (**b**) of LAS and RLAS glasses.

The ^29^Si MAS-NMR spectra of the glasses presented broad peaks ranging from −110 to 80 −200 to 0 ppm (Figure 3a), indicating the Qn groups in the glass structures, Q^4^ groups at −110~−100 ppm, Q^3^ at −95~−90 ppm, Q^2^ at −90~−80 ppm, and Q^1^ −76~−68 ppm [28,29,30]. It is reasonable to propose that the glass was composed of Q^3^ and Q^2^ since the broad peaks were centered at ~90 ppm. Furthermore, the almost overlapped NMR spectra further confirmed the small effect on the glass structure from CaO, SrO, and BaO addition. The peak center of the MLAS glass was slightly shifted to the left by 2 ppm, suggesting that Mg^2+^ acted as a network former and charge compensator and increased the amount of bridging oxygen associated with Si, thus affecting the chemical shift in ^29^Si. The ^27^Al NMR spectra of all glasses exhibited broad asymmetric peaks centered at 51 ppm (Figure 3b), indicating the main [AlO_4_] groups and minor [AlO_5_] and [AlO_6_] groups. The unchanged spectra, with different alkali earth oxide additions, confirmed the compositional indecency of Al coordination groups in the glass structures [31].

### 3.2. The Thermal Properties of the Glass

**Figure 4:**
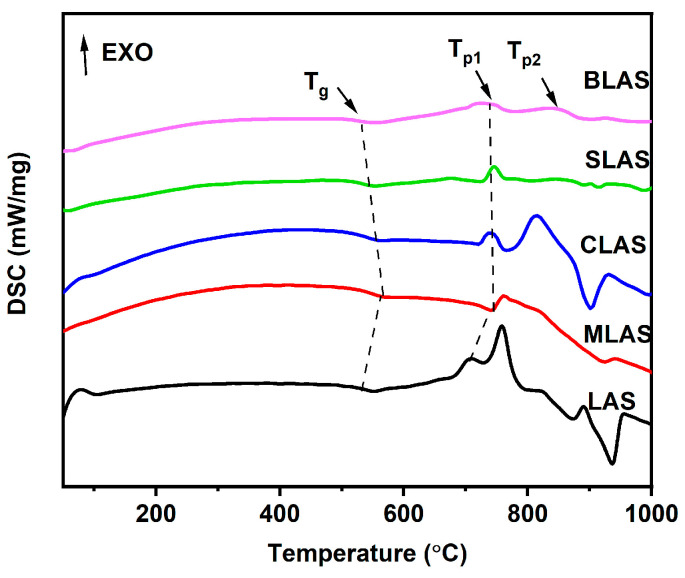
DSC diagram of LAS and RLAS glasses.

The DSC diagrams of the glasses (Figure 4) show that there was an increase in glass transition temperatures (T_g_) from 501.4 °C to 547.3 °C due to MgO addition and then a decrease with greater alkali earth oxide introduction, as listed in Table 1. Meanwhile, the glass crystallization peak temperatures (T_p_) also exhibited a similar trend. The increased T_g_ and T_p_ could originate from the better glass connectivity [16,32] due to the high local electric field and small ionic radius of Mg^2+^ resulting in MgO glass formers in the microstructures, which was consistent with the analysis from the Raman (Figure 2c) and NMR (Figure 3a) spectra. On the other hand, the larger ionic radius of Ca, Sr, and Ba decreased the local electric field, making CaO, SrO, and BaO act as glass modifiers and destroy the glass networks [33]. The differences between T_g_ and T_c_ were larger than 200 °C, implying the good glass-forming abilities of all compositions.

**Figure 5:**
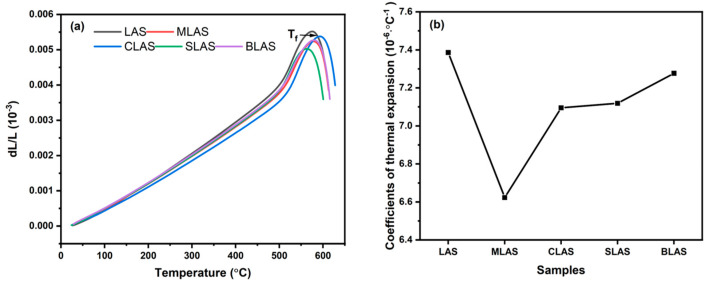
The temperature dependence of the glass rod length change (**a**) and calculated CTE (**b**) of LAS and RLAS glasses.

Asymmetric changes in vibrational amplitude and potential energy between two atoms at elevated temperatures largely determine the coefficient of thermal expansion (CTE) of a glass [34,35]. The temperature dependence of the glass bar length and the calculated CTE values are shown in Figure 5a, b. With the introduction of alkaline earth oxides into the glass, the CTE values gradually increased from 6.23 × 10^−6^/°C to 7.27 × 10^−6^/°C, with a lager ionic radius, from Mg to Ba. In general, the ion field strength decreased with a growing ionic radius, which led to the higher amplitude of the thermal vibration and eventually the easier expansion of the glass. The dropping CTE from LAS to MLAS could be attributed to the possible MgO network formed in the microstructure.

**Figure 6:**
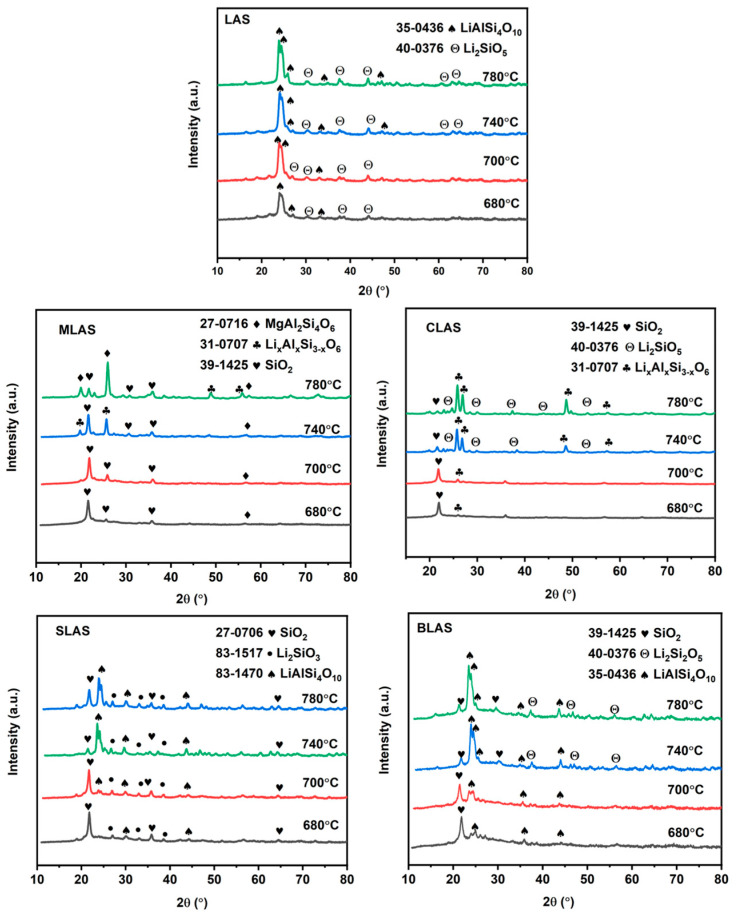
XRD patterns of glass–ceramics: LAS, MLAS, CLAS, SLAS, and BLAS, after heat treatment at 640 °C for 2 h and 680–780 °C for 1 h.

The glasses were heat-treated for crystal precipitation and glass–ceramic formation. The protocol was a two-step heat treatment, with the first step at 640 °C for 2 h for nucleation and different temperatures at 680, 700, 740, and 780 °C for 1 h for crystal growth for all glasses. The XRD patterns (Figure 6) demonstrated the crystallization evolution. All the glasses showed crystal phases after heat treatment in the second step of 680 °C for 1 h. Then, the crystal phases changed and the sizes grew with higher temperatures. For the LAS glass, lithium disilicate (Li_2_Si_2_O_5_), petalite (LiAlSi_4_O_10_), and silica (SiO_2_) crystals were precipitated in the glass matrix. Meanwhile, SiO_2_ crystals formed in the glass matrix with MgO addition in the low-temperature second-step heat treatment, and Li_x_Al_x_Si_3−x_O_6_ crystals appeared with increased temperature; then, MgO was involved in crystal formation and MgAl_2_Si_4_O_12_ crystals were precipitated due to MgO with a further temperature increase. For CLAS, with CaO introduction, SiO_2_ crystals formed at first; then, Li_x_Al_x_Si_3−x_O_6_ crystals appeared, and crystals grew with a further temperature increase, without new Ca-O-related phase formation. For the glasses with SrO and BaO addition, SiO_2_ and Li_2_SiO_3_ crystals formed at low temperatures, and then LiAlSi_4_O_10_ crystals were precipitated and grew, which was similar to the crystal formation and growth procedure in the LAS glass without alkali earth oxides’ introduction. SrO and BaO were not involved in the crystal phases. It is easy to understand that large-radii Ca^2+^, Sr^2+^, and Ba^2+^ ions could not be incorporated into Si-Al-O-related crystal phases [36]. The different crystal phases in the glass matrix implied the different mechanical performances of glass–ceramics.

**Figure 7:**
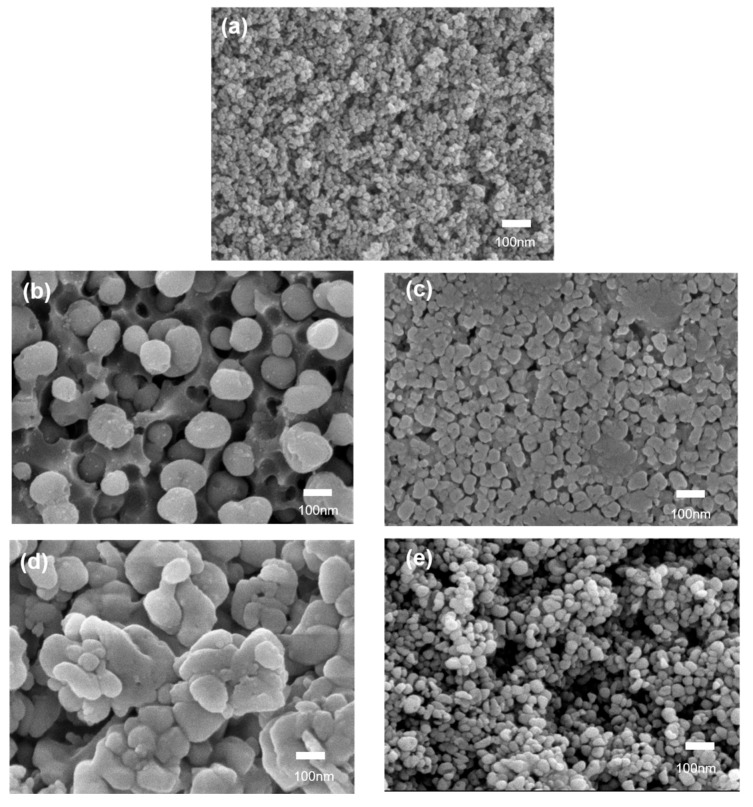
SEM images of nanocrystals in LAS (**a**), MLAS (**b**), CLAS (**c**), SLAS (**d**), and BLAS (**e**) glass–ceramics after two-step heat treatment at 640 °C for 2 h and 780 °C for 1 h.

The crystal morphologies in the glass matrix after the two-step heat treatment, at 640 °C/2 h + 780 °C/1 h, confirmed the uniform distribution of spherical crystal precipitation (Figure 7). The diameters of the crystals were varied with different alkali earth oxides’ introduction, with fine nanocrystals with a diameter around 30 nm for LAS, around 100 nm for MLAS, around 50 nm for CLAS, and 30 nm for BLAS glass–ceramics, indicating different crystal formation and growth processes due to alkali earth oxides’ introduction.

### 3.3. The Mechanical Performances of the Glass and Glass–Ceramics

**Figure 8:**
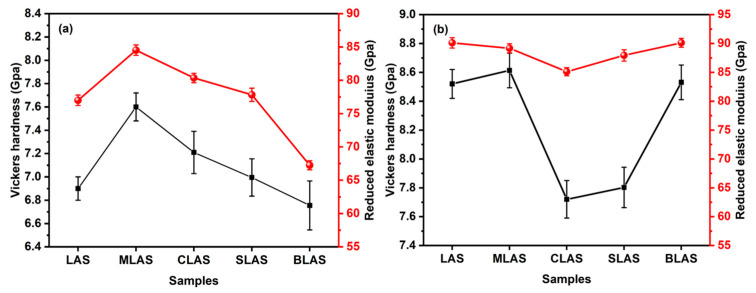
The Vickers hardness and the elastic modulus of the LAS and RLAS glasses (**a**) and glass–ceramics (**b**) after the two-step heat treatment at 640 °C for 2 h and 780 °C for 1 h.

The Vickers hardness and elastic modulus of the as-prepared glass (Figure 8a) increased from 6.91 GPa and 77.2 GPa for the LAS glass, with a maximum of 7.65 GPa and 84.2 GPa with MgO introduction, and then gradually decreased to 6.75 GPa and 67.25 GPa with larger-radii alkai earth oxides’ introduction. The changes in the hardness and modulus probably resulted from the different microstructures of the glass. The Raman spectra of the MLAS glass showed obvious changes from those of LAS, and the MgO in the glass matrix could act as a glass former in a certain range, which increased the glass connectivity. On the other hand, the introduction of CaO could make SrO and BaO glass modifiers, which decreased the glass connectivity. Furthermore, the larger ionic radius decreased the electric field intensities, which further decreased the glass transition and crystallization temperatures (Figure 4 and Table 1) and the mechanical performance. After the two-step heat treatment at 640 °C for 2 h and 780 °C for 1 h, the hardness slightly increased from 8.52 GPa for LAS to 8.61 GPa for MLAS glass–ceramics, dropped to 7.72 GPa for CLAS and 7.82 for SLAS, and recovered to 8.53 GPa for BLAS glass–ceramics (Figure 8b). Meanwhile, the elastic modulus of the glass–ceramics slightly decreased from 90.01GPa for LAS to 89.1 GPa for MLAS and 85.12 GPa for CLAS, and it then increased to 87.93 GPa for SLAS and 90.12 GPa for BLAS. The hardness and modulus values were equivalent to or better than those of other LAS glasses with different modifications (Table 2). The change in the hardness and elastic modulus of the glass–ceramics could originate from crystal phases and crystallinity. The close hardness and elastic modulus between LAS and BLAS could result from similar crystal phases (Figure 6 LAS, BLAS) and morphologies (Figure 7a,e).

**Figure 9:**
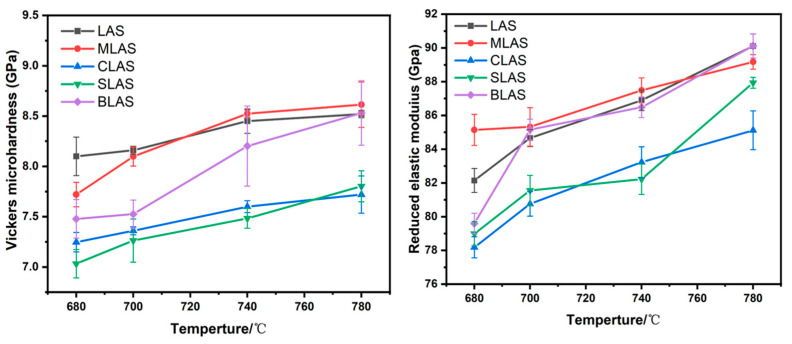
The Vickers hardness and the elastic modulus of the LAS and RLAS glass–ceramics, with different second-step heat-treatment temperatures (GPa).

The Vickers hardness and elastic modulus increased after heat treatment, for all the glass, due to the formation of crystals in the glass matrix (Figure 9). When crystallized at 680 °C, the BLAS glass–ceramic exhibited a Vickers hardness of ~7.56 GPa due to the low crystallinity of ~34.71%. When the crystallization temperature continued to increase to 700 °C, 740 °C, and 780 °C, the crystallinities were ~43.89%, ~60.87%, and ~63.21%, respectively. The Vickers hardness of the BLAS glass–ceramic increased with increasing crystallinity. Meanwhile, the LAS and RLAS glass–ceramic samples had similar trends. Therefore, as the crystallization temperature increased, the crystallinity of the glass and the proportion of the crystalline phase increased, thus increasing the Vickers hardness of the glass–ceramics. The Vickers hardness of BLAS microcrystals at 780 °C was as high as 8.53 GPa, and the elastic modulus was as high as 90.12 GPa. At the same time, the Vickers hardness of LAS microcrystals at 780 °C was as high as 8.52 GPa, and the elastic modulus was as high as 90.01 GPa. The Vickers hardness and modulus were improved due to the crystallization of the glass and were highly dependent on the crystalline phase. It is reasonable to believe that the high hardness and high elastic modulus of BLAS came from the effect of the LiAlSi_4_O_10_ crystal phase.

**Figure 10:**
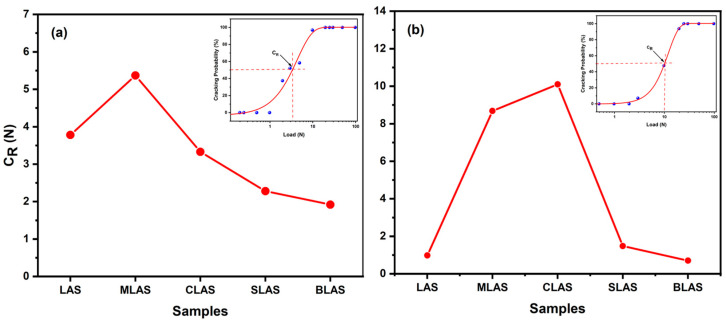
The cracking resistance of LAS and RLAS glasses. (**a**) Inset: the dependence of cracking probabilities on the load of the MLAS glass and glass–ceramics after two-step heat treatment at 640 °C for 2 h and 780 °C for 1 h. (**b**) Inset: the dependence of cracking probabilities on the load of CLAS glass–ceramics.

Crack resistance (C_R_) is the critical indentation load measured by a Vickers indenter and can be used to evaluate the crack germination hindering ability of a glass [37]. In order to evaluate the fracturing resistance of the glass and glass–ceramics, the C_R_ value of the glass and glass–ceramics are shown in Figure 10, along with the relationship between the load value and cracking probabilities of the highest C_R_ value of MLAS for the glass (Figure 10a inset) and CLAS for the glass–ceramics (Figure 10b inset). For the glass (Figure 10a), the C_R_ values rapidly increased to 5.37 N due to MgO introduction and then decreased with other larger alkali earth oxides. Generally, glasses with stronger oxide bonds have higher crack resistance due to higher fracture surface energy [38,39]. The high-field-strength cation (Mg) could form a relatively strong oxidative bond with oxygen and exhibit a high C_R_ due to its high fracture surface energy. Compared with the glass containing Mg, the glass containing Ca, Sr, and Ba had a larger atomic packing density (Figure 2b), which resulted in the decreased densification space and fragmentation resistance of the glass. In fact, the C_R_ values of the MLAS and CLAS glass–ceramics were greatly improved, due to the MgO and CaO in the glass components. This was probably because the main crystal phases in the MLAS sample were MgAl_2_Si_4_O_12_ and Li_x_Al_x_Si_3−x_O_6_, and the main crystal phase in the CLAS sample was Li_x_Al_x_Si_3−x_O_6_. The C_R_ of CLAS being higher than that of the MLAS glass–ceramic was probably due to the formation of the high-expansion-quartz solid-solution MgAl_2_Si_4_O_12_ phase, which produced certain stress and reduced the anti-cracking ability. Meanwhile, the LAS, SLAS, and BLAS glass–ceramics showed decreased C_R_ due to crystallization.

**Table 2:** Mechanical properties and crystalline phases of LAS microcrystalline glasses with different compositions.

Composition System	Crystallization Process	Main Crystalline Phases	Vickers Hardness (GPa)	Elastic Modulus (GPa)	References
LAS	680–750 °C/1–2 h + 800–950 °C/1–4 h	β-quartz, β-Spodumene	6.5–7.5	95–110	[40]
LAS + TiO_2_	720 °C/0.5 h + 850 °C/1 h	β-quartz	6.8–7.3	80–90	[41]
LAS + ZrO_2_	700 °C/1 h + 880 °C/1.5 h	β-quartz	7.2–7.6	105–112	[42]
MLAS	640 °C/2 h + 780 °C/1 h	MgAl_2_Si_4_O_12_, LixAlxSi_3−x_O_6_	8.61	89.1	This work

## 4. Conclusions

In conclusion, different alkali earth oxide-modified LAS glasses and glass–ceramics were prepared, and the density and molar volume of the glasses increased gradually with the increase in the alkaline earth oxide cation radius. The glass containing BaO showed the highest density and molar capacity, with a density of 2.67 g/cm^3^ and a molar volume of 24.21 cm^3^/mol. The introduction of MgO into the glass had an obvious effect on the microstructures, and Mg^2+^ had both glass formation and modifier roles in the glass, while CaO, SrO, and BaO introduction had weak influences. The glass transition and crystallization temperatures increased with MgO and then decreased with CaO, SrO, and BaO. Meanwhile, the CTE of the glass dropped due to MgO introduction and then increased, due to the decreasing electrical field intensities of alkali earth oxides with larger ionic radii. After heat treatment, different crystal phases were precipitated in the glass matrix, including Li_2_Si_2_O_5_, LiAlSi_4_O_10_, and SiO_2_ in the LAS glass, Li_x_Al_x_Si_3−x_O_6_, MgAl_2_Si_4_O_12_, and SiO_2_ in the MLAS glass, Li_x_Al_x_Si_3−x_O_6_ and SiO_2_ in the CLAS glass, and Li_2_SiO_3_ and LiAlSi_4_O_10_ in the SLAS glass, and similar crystal phases were precipitated in the BLAS glass and LAS glass. The evolution of the crystallization behaviors led to the different mechanical performances of the glass–ceramics. The introduction of MgO improved the hardness, which reached a maximum 8.61 GPa, while the elastic modulus reached a maximum of 89.1 GPa in the LAS glass–ceramics. Moreover, the crack resistance values showed a maximum for the MLAS glass and for the CLAS glass–ceramics, due to the glass structure and crystal phase change. This research can provide promising candidates for high-strength glass and glass–ceramics.

## Figures and Tables

**Table 1:** The glass transition temperature (T_g_), the first crystallization peak temperature (T_p1_), and the second crystallization peak temperature (T_p2_) of LAS and RLAS glasses obtained from the DSC diagram.

	LAS	MLAS	CLAS	SLAS	BLAS
T_g_ (°C)	501.4	547.3	534.3	529.7	528.4
T_p1_ (°C)	708.5	741.4	738.7	687.8	685.9
T_p2_ (°C)	758.3	827.7	814.2	734.0	731.2

## Data Availability

The data presented in this study are available on request from the corresponding author. The data are not publicly available due to privacy.
